# Co-culturing bacteria and microalgae in organic carbon containing medium

**DOI:** 10.1186/s40709-016-0047-6

**Published:** 2016-04-26

**Authors:** Jichang Han, Lin Zhang, Song Wang, Guanpin Yang, Lu Zhao, Kehou Pan

**Affiliations:** Laboratory of Applied Microalgae Biology, Ocean University of China, Qingdao, 266003 China; Key Laboratory of Marine Biotechnology, Ningbo University, Ningbo, 315211 China; College of Marine Life Sciences, Ocean University of China, Qingdao, 266003 China; Function Laboratory for Marin Fisheries Science and Food Production Processes, Qingdao National Laboratory for Marine Science and Technology, Qingdao, 266237 China

**Keywords:** Microalgae, Bacteria, Microalgae-bacteria co-culture, Mixotrophy

## Abstract

**Background:**

Microalgae frequently grow in natural environment and long-term laboratory cultures in association with bacteria. Bacteria benefit the oxygen and extracellular substances generated by microalgae, and reimburse microalgae with carbon dioxide, vitamins and so on. Such synergistic relationship has aided in establishing an efficient microalga-bacterium co-culturing mode. Obviously, the mutually beneficial relationship can be strengthened with the increase of the densities of microalgae and bacteria. However, nearly all of the early co-cultures were performed under photoautotrophic conditions, thus both microalgae and bacteria were at relatively low densities. In this study, the feasibility of bacteria-microalgae co-cultured under mixotrophic conditions was studied.

**Results:**

Firstly, bacteria mingled with xenic microalgae were isolated and identified based on their 16S rRNA gene sequence (16S rDNA hereafter). Then, the two most frequently found strains of *Muricauda* sp. were co-cultured with axenic microalga (*Tetraselmis chuii*, *Cylindrotheca fusiformis* and *Nannochloropsis gaditana*) in extra organic carbon containing medium. At the end of a co-culture period of 33 days, we found that the final cell density of *T. chuii* and *C. fusiformis* of various treatments was remarkably higher than that of controls (21.37–31.18 and 65.42–83.47 %, respectively); on the contrary, the growth of *N. gaditana* was markedly inhibited. During the co-culture of bacteria with *C. fusiformis*, the cell density of two strains of bacteria firstly decreased, then increased and maintained at a relatively steady level. However, the cell density of bacteria performed a sustaining downward trend when they were co-cultured with *T. chuii* and *N. gaditana*.

**Conclusions:**

Our findings proved that microalgae-bacteria co-cultures under mixotrophic conditions are quite effective strategy for microalgal cultivation.

## Background

Microalgae and their valuable metabolites have the potential to be used as pollution control, food and feed additives, cosmetic, medicine production, etc. [1–3]. Moreover, microalgae are also considered to be the most appropriate feedstock of biofuel production. Diverse studies have been carried out to improve the efficiency of microalgal cultures (e.g., designing new photobioreactor, optimizing culture condition and reforming culture mode) and increase the content of high value substances (e.g., polyunsaturated fatty acid and neutral lipid) [4–9].

Microalgae and bacteria inhabit together almost all aquatic environments and play crucial roles in nutrient cycling and energy flowing. As documented early, bacteria highly influence the growth of microalgae under autotrophic conditions either positively or negatively [10–14]. In general, bacteria promote microalgal growth by (among others) reducing dissolved oxygen concentration, consuming the organic materials excreted by algae [15] and secreting biotin, cobalt amine and thiamine [16]. In turn, microalgae reimburse bacteria with oxygen and extracellular compounds. Such reciprocity implies that microalgal growth can be enhanced by specific bacteria [10, 11].

Many microalgae can grow on light and chemical energy concurrently achieving extraordinarily high growth rate [17–21]. It is imaginable that the bacteria will compete with microalgae for nutrients, either organic or inorganic, when they are added into the mixotrophic cultivation system of microalgae. However, the competition can be abated by adding excessive nutrients or continuously adding nutrients. In other words, the reciprocity between microalgae and bacteria may still exist in such a co-culture system as was documented between bacteria and microalgae/cyanobacteria in wastewater treatment [3] and between probiotics and animals [22].

*Tetraselmis chuii, Cylindrotheca fusiformis* and *Nannochloropsis gaditana* are able to grow under mixotrophic conditions and can be cultured on a large scale [23–25]. Aiming to select bacteria that can promote microalgal growth, and to determine the feasibility of bacterium-microalga co-culture mode in organic carbon containing medium, we isolated and identified bacteria from xenic microalgal culture, and co-cultured the two most abundant bacteria with these three axenic microalgae each in medium containing organic substances. The growth performances of microalgae and bacteria were investigated during a co-culture period of 33 days, and the feasibility of such cultivation strategy was discussed.

## Results

### Isolation and identification of bacteria

In total, 43 bacterial strains were isolated from 16 xenic microalgal strains and they were assigned to 19 genera. Most of them were identified in classes Bacteroidetes, Flavobacteria, α-proteobacteria, and γ-proteobacteria (Table 1). Though these xenic microalgae have been domesticated in laboratory for many years, their bacterial communities were still similar to those of marine environments [26]. The bacterial species in association with different microalgae varied to a relatively large extent suggesting a possible interaction between specific microalgae and bacteria (Table 1) [27, 28]. *Muricauda* sp. which co-existed with 13 of 16 microalgal strains, was found to be the most ubiquitous bacteria (Table 2). All 16 xenic microalgal strains grew well in long-term laboratory culture. As a result, it was inferred that the ubiquitous bacteria survived together with most microalgae were possible to promote microalgal growth. Accordingly, two strains of *Muricauda* sp., Mur1 [GenBank: KM23334] and Mur2 [GenBank: KM23335], were chosen to co-culture with the three selected microalgae.

**Table 1:** Bacterial species isolated from mixotrophically cultured microalgae

Closest relative	Strains	Similarity (%)	E-value	Acc. no.	Classes
*Muricauda* sp.	Mur1	99	0.0	JN594619	Bacteroidetes
	Mur2	99	0.0	KF724486	Bacteroidetes
	Mur3	99	0.0	JN594619	Bacteroidetes
	Mur4	99	0.0	JN594619	Bacteroidetes
	Mur5	99	0.0	JN594619	Bacteroidetes
	Mur6	99	0.0	JN594619	Bacteroidetes
	Mur7	100	0.0	EU839357	Bacteroidetes
	Mur8	99	0.0	AY576776	Bacteroidetes
	Mur9	99	0.0	EU839357	Bacteroidetes
	Mur10	99	0.0	EU839357	Bacteroidetes
*M. aquimarina*	Mur11	100	0.0	KC534238	Bacteroidetes
	Mur12	99	0.0	KF500394	Bacteroidetes
	Mur13	100	0.0	KF500394	Bacteroidetes
	Mur14	99	0.0	KF500397	Bacteroidetes
	Mur15	99	0.0	NR_042909	Bacteroidetes
*Bacteroidetes bacterium*	Bac1	99	0.0	AY162097	Flavobacteria
	Bac2	99	0.0	GU565603	Flavobacteria
*Flavobacterium* sp.	Fla1	100	0.0	AF386740	Flavobacteria
*Aestuariibacter* sp.	Aes1	99	0.0	JF309276	γ-proteobacteria
*Marinobacte*r sp.	Mar1	100	0.0	AB758589	γ-proteobacteria
	Mar2	99	0.0	AM944524	γ-proteobacteria
*M. flavimaris*	Mar3	100	0.0	AB617558	γ-proteobacteria
*M. hydrocarbonoclasticus*	Mar4	99	0.0	JQ799112	γ-proteobacteria
*M. radhaerens*	Mar5	100	0.0	NR_074765	γ-proteobacteria
*Alteromonas* sp.	Alt1	99	0.0	AB636144	γ-proteobacteria
	Alt2	99	0.0	AB636144	γ-proteobacteria
*Pseudoalteromonas* sp.	Pse1	98	0.0	GQ495024	γ-proteobacteria
	Pse2	99	0.0	GQ495024	γ-proteobacteria
*Nitratireductor* sp.	Nit1	99	0.0	JN942153	α-proteobacteria
	Nit2	99	0.0	JN942153	α-proteobacteria
*Stappia* sp.	Sta1	100	0.0	JF899875	α-proteobacteria
*Stappia indica*	Sta2	99	0.0	AB607882	α-proteobacteria
*Labrenzia aggregata*	Lab1	99	0.0	AB681109	α-proteobacteria
*Maricaulis parjimensis*	Mac1	99	0.0	NR_025323	α-proteobacteria
*Zhangella mobilis*	Zha1	99	0.0	EU255260	α-proteobacteria
*Oceanicaulis* sp.	Oce1	100	0.0	AB681546	α-proteobacteria
*Sagittula* sp.	Sag1	99	0.0	KC534267	α-proteobacteria
*Tropicibacter* sp.	Tro1	98	0.0	KC534265	α-proteobacteria
*Rhodopirellula baltica*	Rho1	100	0.0	JN694985	Planctomycetacia
	Rho2	99	0.0	HQ845537	Planctomycetacia
*Cytophaga* sp.	Cyt1	98	0.0	AB073564	Sphingobacteria
*Kocuria rosea*	Koc1	99	0.0	JN192402	Actinobacteria
*Bacillus jeotgali*	Bai1	99	0.0	JX094165	Firmicutes

Acc. no., accession number of GenBank

**Table 2:** Bacteria and corresponding microalgae

Bacterial species	1	2	3	4	5	6	7	8	9	10	11	12	13	14	15	16
*Muricauda* sp.	+			+	+	+	+	+	+	+	+	+		+	+	+
*Bacteroidetes bacterium*		+		+	+		+	+		+	+	+	+	+		+
*Flavobacterium* sp.	+	+				+		+	+			+				
*Aestuariibacter* sp.				+							+				+	
*Marinobacte*r sp.	+				+	+	+		+	+			+			
*Alteromonas* sp.		+										+	+	+		
*Pseudoalteromonas* sp.	+		+			+		+	+							+
*Nitratireductor* sp.			+	+	+			+		+	+		+		+	+
*Stappia* sp.				+	+		+					+	+			+
*Labrenzia aggregata*		+	+				+								+	
*Maricaulis parjimensis*				+	+	+					+	+	+	+		+
*Zhangella mobilis*														+		
*Oceanicaulis* sp.	+	+						+	+			+				
*Sagittula* sp.						+						+		+	+	
*Tropicibacter* sp.											+		+			+
*Rhodopirellula baltica*												+		+		
*Cytophaga* sp.												+	+		+	
*Kocuria rosea*														+	+	+
*Bacillus jeotgali*											+			+	+	

Numbers from 1 to 16 stand for the xenic microalgal strains used in this study, 1: *Nannochloropsis oceanic*; 2: *N. gaditana*; 3: *N. oculata*; 4: *Thalassiosira* sp.; 5: *Chaetoceros gracilis*; 6: *Navicula* sp.; 7: *Cylindrotheca fusiformis*; 8: *Phaeodactylum tricornutum*; 9: *Pseudo*-*nitzschia* sp.; 10: *Pavlova* sp.; 11: *Chromulina* sp.; 12: *Gymnodinium* sp.; 13: *Amphidinium carterae*; 14: *Prorocentrum minimum*; 15: *Karenia mikimotoi*; 16: *Heterosigma carterae*

+, bacterium was obtained; margin, bacterium was not isolated

### Growth curves of Mur1 and Mur2

**Fig. 1:**
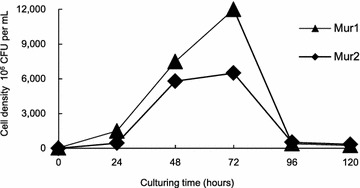
Growth curves of Mur1 and Mur2. *Error bars* are too small to be seen

The density of Mur1 and Mur2 each maximized at 72 h (1.2 × 10^10^ and 6.5 × 10^9^ CFU mL^−1^, respectively), then drastically declined to 4.0 × 10^8^ and 5.2 × 10^8^ CFU mL^−1^ at 96 h (Fig. 1) suggesting that the bacteria after reaching the maximal density might begin to secrete extracellular substances, which would affect the microalgal growth. As a result, Mur1 and Mur2 cultured for 24 h, with relatively low concentration and high metabolic activity, were determined for use in the following co-culture phase.

### Detection of extra bacterial contamination

No different bacterial colony was observed on the plates, and the sequencing results of co-inoculated samples were consistent with that of Mur1 or Mur2 indicating that all treatments have not been contaminated by external bacteria.

### Growth performance of bacterium during co-culture

**Fig. 2:**
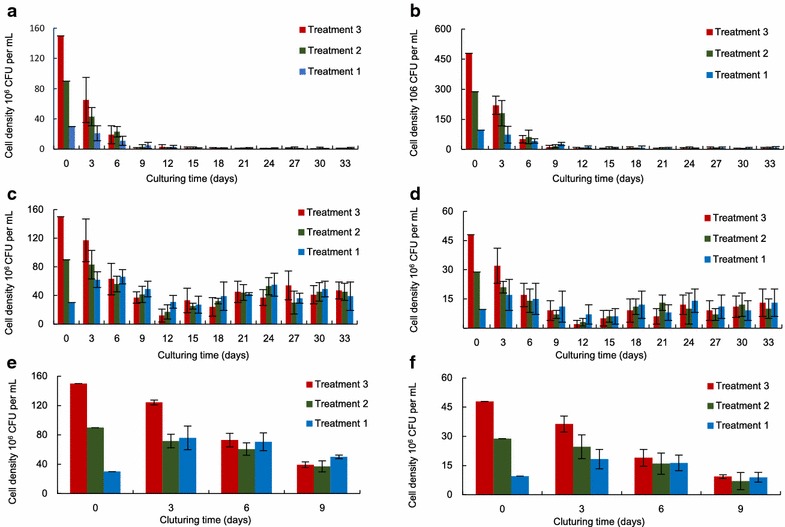
Growth performances of Mur1 and Mur2 during co-culture. Mur1 and Mur2 co-cultured with *T. chuii (* **a,** **b**); *C. fusiformis* (**c**, **d**); *N. gaditana* (**e**, **f**), respectively

For the combinations of Mur1, Mur2 and *T. chuii*, the bacterial densities of all treatments have drastically reduced in first 9 days, and remained at relatively low levels since then. Despite the initial density of different treatments varied greatly, their final density was similar to each other (Fig. 2a, b).

In co-culturing Mur1 and *C. fusiformis*, the bacterium of treatment one presented a transient increase in first 6 days, decreased to the minimum on the day 15, then displayed a slight increase and maintained at a relatively stable state (around 4.5 × 10^7^ CFU mL^−1^).The variation of bacterial density of treatment two and three was similar to each other; both decreased firstly, and then increased to a relatively stable amount (around 4.5 × 10^7^ CFU mL^−1^). The overall variation trend of three treatments of Mur2 was quite similar to those of Mur1 (Fig. 2c, d).

The cell density of bacterium added into *N. gaditana* has been traced for 9 days. As shown in Fig. 2e and f, the variation trends of Mur1 and Mur2 was quite similar to those of *C. fusiformis*.

### Growth performances of microalgae during co-culture

**Fig. 3:**
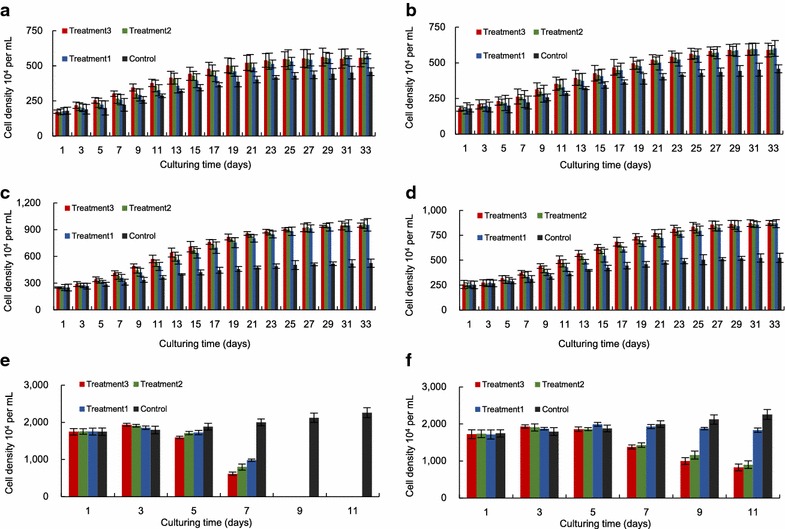
Performance of three microalga during co-culture. *T. chuii* (**a,** **b**), *C. fusiformis* (**c,** **d**) and *N. gaditana* (**e,** **f**) co-cultured with Mur1 and Mur2

As shown in Fig. 3, the growth performance of three microalga changed greatly during co-culture. Mur1 and Mur2 promoted the growth of *T. chuii* and *C. fusiformis* heavily, but inhibited the growth of *N. gaditana* drastically.

The final cell density of *T. chuii* co-cultured with Mur1 or Mur2 was higher than that of control. Three treatments co-cultured with Mur1 increased the final cell density of *T. chuii* by 21.37 to 23.91 % (the highest was 5.68 × 10^6^ cells mL^−1^). For Mur2, the improvement ranged from 28.63 % to 31.18 % (the highest was 6.01 × 10^6^ cells mL^−1^) (Fig. 3a, b). The enhancement of Mur1 and Mur2 to the growth of *C. fusiformis* was much greater than that to *T. chuii*. When co-cultured with Mur1 and Mur2, the cell density of *C. fusiformis* increased by about 80 and 65 % over control, respectively (the highest cell density was 9.62 and 8.73 × 10^6^ cells mL^−1^, respectively) (Fig. 3c, d).

Such promotion was not found in *N. gaditana*. Instead of enhancement, the growth of *N. gaditana* of all treatments was obviously inhibited by Mur1 and Mur2 (Fig. 3e, f). The inhibitory effect of Mur1 on *N. gaditana* was greater than that of Mur2. Microscopic observation showed that some cells of *N. gaditana* started to rupture on day 5.

## Discussion

In the present study, the positive effect of selected bacteria on the growth of microalgae was observed, indicating the co-culture mode of microalgae-bacteria in the organic substance containing medium was feasible and highly efficient. On the other hand, the results of the present study indicated that specific bacteria can drastically inhibit the growth of specific microalgae, as in the case of *N. gaditana*. The phenomenon of specific bacterium induce different effect on different microalgae has been reported earlier [29, 30], which indicated that the determination of suitable combination of microalga and bacterium was quite important for the successful implementation of co-culture mode.

In general, bacterial metabolism can benefit microalgal growth by providing favorable microenvironments, e.g., increasing carbon dioxide, secreting vitamins and reducing photosynthetic oxygen tension [16, 31, 32]. Obviously, such beneficial effects will be enhanced by increasing bacterial density as it has been demonstrated by the results of the present study. For example, the higher the initial concentration of Mur1, the faster the growth of *C. fusiformis* in the early phase of co-culture (day 2–8). By consuming the original organic substance, the bacterial density of all treatments reduced and maintained at a similar level, which further resulted in a similar microalgal growth rate and a final microalgal density among all treatments. Such observation is in contrast to previous reports [33]. The relatively sufficient nutrient in our culture system may have alleviated the competition between microalga and bacterium, thus being responsible for this difference.

Except for positive effect, drastically inhibition phenomenon was also observed. Overall, the bacterium can restrain the microalgal growth by several ways including (among others) the competition for nutrients [34–36], the excretion of algicidal substances [37–39], and the degradation of the cell wall of the microalgal cell through direct contact [40–42]. For the combination of Mur1/Mur2 and *N. gaditana*, several phenomena can be detected. For example, the microalgal density of all treatments was obviously lower than that of control except at day 3; the inhibitory effect of bacteria on *N. gaditana* enhanced with the increase of the original bacterial concentration; the bacterial densities of all groups except the combinations of *N. gaditana* and Mur1/Mur2 at the ratios of 1.96/0.59 declined all the time; and the cells of *N. gaditana* began to rupture after 5 days of co-culture. As a result, it can be inferred that the inhibitory effect was caused by direct contact, and such effect may appear as long as the density ratio of bacterium to microalgae reached a certain threshold.

During co-culture, not only the density of microalgae changed greatly, but also the concentration of bacteria experienced large fluctuations. Following the consumption of the original organic substance, the concentration of bacterium of nearly all treatments shared a common declining trend in first 9 days. As the incubation time prolonged, the variation trend of bacterial density among different combinations began to diverge, either continuously declining (*T. chuii*) or steadily rising (*C. fusiformis*). Such obvious discrepancy can come down to the difference of excreting extracellular substances between a benthic diatom and a green alga. In addition, the compounds secreted by *T. chuii* may also play a role on the continuous decline of bacterial density as it has been observed in other microalgae [43–45].

Based on our results, it can be deduced that bacteria and microalgae interacted each other when they were co-cultured in the medium containing extra organic substance. Screening bacteria capable of promoting microalgal growth is relatively simple. However, it needs more attention to determine an optimum candidate for a specific microalga. For example, the final density of *C. fusiformis* was around 80 and 65 % higher than that of control when it was co-cultured with Mur1 and Mur2, respectively; however, the promotion became around 23 and 30 % for *T. chuii*. This difference was possibly caused by different performances of bacteria and microalgae in the ability of competing for nutrients (both organic and inorganic), the respiratory and photosynthetic rate and the kind and amount of excreting substance.

## Methods

### Microalgal strains

Sixteen xenic microalgal strains (Table 2) were obtained from the culture collection of microalgae of Ocean University of China, and cultured in f/2 seawater medium [46] at 25 ± 1 °C and under 70 µmol photons m^−2^ s^−1^ irradiation with a photoperiod of 12 h light and 12 h dark. Three axenic microalgae (*T. chuii*, *C. fusiformis* and *N. gaditana*) were obtained as described previously [47]. The axenic algal cells stained by SYBR Green I (Solarbio, China) were examined under fluorescence microscope [47, 48].

### Isolation and preliminary identification of bacteria

Microalgae in stationary growth phase were inoculated into fresh f/2 medium at a ratio of 1/10 (v/v) and cultured for 20 days. One mL of microalgal culture was 1000 fold diluted with sterile f/2 medium. Then, 1 mL of the dilution was mixed with 25 mL of sterile 2216E marine bacterium solid culture medium (1.5 % agar containing), poured into Petri dish and cultured at 37 °C for 72 h. In total, 15 colonies were picked up from each plate, and inoculated into 2 mL of 2216E liquid medium and cultured at 37 °C and 200 rpm for one day. After that, the culture was streaked and cultured for 3 days. A single colony was picked up and inoculated into 2 mL of 2216E medium and cultured for 2 days. One mL of each culture was stored at −80 °C in 20 % of glycerol (v/v) and the rest 1 mL was used to extract DNA with CTAB method [49]. The universal primers for the amplification of 16S rRNA gene used in this study were listed in Table 3. In this procedure, primers of 27F and 1492R were preferred, and the pairs of 63F and WBAC2, E9F and 1542R were taken as alternatives.

**Table 3:** Primers used for the amplification and sequencing of 16S rRNA

Primers	Sequences (5′-3′)	References
27F	AGAGTTTGATCCTGGCTCAG	[50]
1492R	CGGCTACCTTGTTACGACTT	[50]
63F	CAGGCCTAACACATGCAAGTC	[51]
WBAC2	CCCGGGAACGTATTCACCGCG	[51]
E9F	GAGTTTGATCCTGGCTCAG	[52]
1542R	AGAAAGGAGGTGATCCARCC	[53]

PCR was carried out on a thermocycler (Eppendorf, Germany) with a cycling regime of (1) one cycle of 5 min at 94 °C, (2) 30 cycles of each 30 s at 94 °C, 30 s at 50 °C, and 1.5 min at 72 °C, and (3) 10 min at 72 °C. The final volume of PCR mix was 50 µL containing 25 µL *EasyTaq*^®^ PCR SuperMix (TransGen Biotech, Beijng, China), 1 µL of each primer (0.2 µM), 2 µL DNA template and 11 µL ddH_2_O. PCR products were purified with OMEGA Gel Extraction Kit (OMEGA, USA) and subsequent samples were sent to Sango Company (Shanghai, China) for sequencing.

### Primary determination of growth curves of Mur1 and Mur2

Many bacteria secrete extracellular substances after reaching their maximum densities [54]. To reduce the effects of such potential substances on microalgal growth as much as possible, the growth curves of the two most abundant bacterial strains, Mur1 and Mur2, were drawn ahead of co-culture.

One hundred μL of bacterial culture maintained at −80 °C were transferred to 400 mL of 2216E medium, cultured at 37 °C and 200 rpm for 120 h. During this period, one mL of culture was taken every day and it was tenfold diluted. Then, 1 mL of the dilution was mixed with 25 mL of 2216E marine bacterium solid medium and cultured under the same conditions as above for 72 h with colonies counted. Plates with 20–200 colonies were chosen to calculate the bacterial density using the equation: bacterial density (CFU mL^−1^) = colony number × dilution fold.

### Algae-bacteria co-culture

Four mL of axenic microalgae each in stationary growth phase, in which the cell density of *T. chuii*, *C. fusiformis* and *N. gaditana* was about 2.0 × 10^6^, 2.8 × 10^6^ and 1.9 × 10^7^ cells mL^−1^, respectively, were inoculated into 41 mL of fresh f/2 medium and cultured at 25 ± 1 °C and under 70 µmol photons m^−2^ s^−1^ irradiation under a light cycle of 12 h light and 12 h dark for 15 days. The final cell density was determined with a hemocytometer (Qiujing, Shanghai, China).

One hundred μL of Mur1 and Mur2 maintained at −80 °C were inoculated into 400 mL of fresh 2216E medium and cultured at 37 °C and 200 rpm for 24 h. The bacterial density was determined with the method described above.

Afterwards, 1 mL (treatment 1), 3 mL (treatment 2), and 5 mL (treatment 3) of the bacteria were collected by centrifugation and suspended in 5 mL modified 2216E medium (1 L seawater containing 5.0 g peptone, 1.0 g yeast extract, 1.0 g glucose and 0.1 g FePO_4_), mixed with 45 mL of axenic microalgal culture. Microalgae each mixed with 5 mL of modified 2216E medium were cultured as controls. The microalgae/bacteria density was investigated every 2/3 days respectively, with the method described above. The initial density ratios of bacteria to microalgae of various treatments were listed in Table 4.

**Table 4:** Initial ratios of bacterial to microalgal cell numbers

Ratios	Treatment 1	Treatment 2	Treatment 3
Mur 1 : *T. chuii*	19.61	58.82	98.04
Mur 1 : *C. fusiformis*	13.33	40.00	66.67
Mur 1 : *N. gaditana*	1.96	5.88	9.80
Mur 2 : *T. chuii*	5.88	17.65	29.41
Mur 2 : *C. fusiformis*	4.00	12.00	20.00
Mur 2 : *N. gaditana*	0.59	1.76	2.94

### Verification of bacterial contamination

On the last day of co-culture, 1 mL of 1000-fold dilution of microalgal culture was mixed with 2216E solid medium and cultured under the same conditions as above for 3 days. Thirty bacterial colonies were picked from each plate randomly and co-inoculated into 2 mL of 2216E and cultured for 2 days. The 16S rDNA was amplified with primer 27F and 1492R, sequenced and used to perform a local alignment (Bioedit) with the sequences of Mur1/Mur2.

## Conclusions

Compared to autotrophic culturing system, additional organic nutrients in our co-culturing system yielded higher cell densities of both microalgae and bacteria. As a result, the effect of bacteria on microalgae, either positive or negative, was heavily intensified. Such co-culture mode was quite different from those documented early displaying obvious enhancements on the growth of *T. chuii* and *C. fusiformis*, which was potential to be used in microalgal cultivation.
